# Seeking for Correlative Genes and Signaling Pathways With Bone Metastasis From Breast Cancer by Integrated Analysis

**DOI:** 10.3389/fonc.2019.00138

**Published:** 2019-03-13

**Authors:** Yu Zhang, Wendan He, Sen Zhang

**Affiliations:** ^1^Department of Orthopaedics, The First People's Hospital of Chengdu, Chengdu, China; ^2^Department of Stomatology, Shenzhen Hospital, Southern Medical University, Shenzhen, China; ^3^State Key Laboratory of Bioactive Substances and Functions of Natural Medicines, Institute of Materia Medica, Chinese Academy of Medical Sciences & Peking Union Medical College, Beijing, China

**Keywords:** breast cancer, bone metastasis, diagnose, mRNA expression profile, protein-protein interaction, transcription factors-target gene

## Abstract

**Background:** Bone metastasis frequently occurs in advanced breast cancer patients, and it is one of major causes of breast cancer associated mortality. The aim of the current study is to identify potential genes and related signaling pathways in the pathophysiology of breast cancer bone metastasis.

**Methods:** Three mRNA expression datasets for breast cancer bone metastasis were obtained from Gene Expression Omnibus (GEO) dataset. The differentially expressed genes (DEGs) were obtained. Functional analyses, protein-protein interaction (PPI) network, and transcription factors (TFs)-target genes network was constructed. Real-time PCR using clinical specimens was conducted to justify the results from integrated analysis.

**Results:** A 749 DEGs were obtained. Osteoclast differentiation and rheumatoid arthritis were two significantly enriched signaling pathways for DEGs in the bone metastasis of breast cancer. SMAD7 (degree = 10), TGFBR2 (degree = 9), VIM (degree = 8), FOS (degree = 8), PDGFRB (degree = 7), COL5A1 (degree = 6), ARRB2 (degree = 6), and ITGAV (degree = 6) were high degree genes in the PPI network. ETS1 (degree = 12), SPI1 (degree = 12), FOS (degree = 10), FLI1 (degree = 5), KLF4 (degree = 4), JUNB (degree = 4), NR3C1 (degree = 4) were high degree genes in the TFs-target genes network. Validated by QRT-PCR, the expression levels of IBSP, MMP9, MMP13, TNFAIP6, CD200, DHRS3, ASS1, RIPK4, VIM, and PROM1 were roughly consistent with our integrated analysis. Except PROM1, the other genes had a diagnose value for breast cancer bone metastasis.

**Conclusions**: The identified DEGs and signaling pathways may make contribution for understanding the pathological mechanism of bone metastasis from breast cancer.

## Introduction

Breast cancer, a complex and heterogeneous disease, is the leading cause of cancer-associated death in the women worldwide (1). In China, breast cancer exhibits one of the fastest growing incidence of cancer, which has become the leading cause of mortality in females (2). Clinically, metastasis is a major challenge that leads to 80% of cancer-associated death (3). It is worth mentioning that most patients with breast cancer die from tumor metastases. It is found that bone is one of the most frequent metastatic site for breast cancer, with about 70% of patients with advanced breast cancer exhibiting bone metastasis (4). Furthermore, bone micrometastases are present in approximately 30% of breast cancer patients diagnosed with stage I, II, and III (5). These breast cancer patients with bone metastasis also have substantial complications, including pain, hypercalcemia, and increased risk of fracture (6).

It is reported that the pathology of bone metastasis is characterized by a vicious cycle where the balance between bone forming cells “osteoblasts” and the bone resorbing cells “osteoclasts” is imbalanced (7). For women with early breast cancer, younger age, primary tumor size >2 cm, estrogen receptor positive/progesterone receptor negative, the presence of significant nodal disease are risk factors for developing bone metastases (8, 9). Generally, it is incurable except palliative therapies when the tumor has metastasized to bone (10). Although significant progress in long—term survival has been made, early diagnosis, prognosis of breast cancer remain poor (11). Therefore, identifying potential pathological genes and signaling pathways influencing the bone metastasis of breast cancer process is very important.

In this study, we aimed to find differentially expressed genes (DEGs) in breast cancer bone metastasis by integrated analysis. Then, functional enrichment analysis including Gene Ontology (GO) and the Kyoto Encyclopedia of Genes Genomes (KEGG) was used to investigate the biological function of DEGs. Protein-protein interaction (PPI) and transcriptional factors (TFs)-target genes network construction was performed to further investigate the function of identified DEGs. Quantitative reverse transcription-polymerase chain reaction (QRT-PCR) was applied to validate the expression of candidate DEGs. Finally, receiver operating characteristic (ROC) analyses was applied to analyze diagnosis ability of identified DEGs. Our study may be helpful in understanding the pathogenic mechanism of bone metastasis from breast cancer, and most difference from previous studies is that current study focuses on bone metastasis of breast cancer and aim to find the differential expressed genes and pathways from other site metastasis of breast cancer.

## Materials and Methods

### Datasets

In this study, we searched datasets of breast cancer bone metastasis from the Gene Expression Omnibus (GEO) database (http://www.ncbi.nlm.nih.gov/geo/) with the keywords (“breast neoplasms” [MeSH Terms] OR breast cancer [All Fields]) AND (“bone and bones” [MeSH Terms] OR bone [All Fields]) AND “Homo sapiens”[porgn] AND “gse”[Filter]. The dataset will be screened for the integrated analysis; (1) The study type is “expression profiling by array.” (2) All selected datasets were from breast cancer metastasis of bone and/other sites metastasis of tumor samples. (3) The standardized or primary datasets were included in this study. Finally, a total of 3 datasets including GSE14020, GSE54323, and GSE46141 was screened, which was shown in Table 1.

**Table 1:** Three datasets of selected breast cancer with bone metastasis.

**Datasets**	**Platforms**	**Other sites metastasis**	**Bone metastasis**	**Year**	**Country**	**PMID**
GSE14020	GPL96 [HG-U133A] Affymetrix Human Genome U133A Array	28	8	2009	USA	19573813; 25670079
	GPL570[HG-U133_Plus_2] Affymetrix Human Genome U133 Plus 2.0 Array	19	10			
GSE54323	GPL570[HG-U133_Plus_2] Affymetrix Human Genome U133 Plus 2.0 Array	15	14	2015	Sweden	25888067
GSE46141	GPL10379 Rosetta/Merck Human RSTA Custom Affymetrix 2.0 microarray [HuRSTA-2a520709]	86	5	2013	Sweden	24287398

### Identification of DEGs

Raw expression data in this study was downloaded. Limma and Meta-analysis for MicroArrays (metaMA) packages were used to identify the DEGs, and the inverse normal method was used to combine the *p*-value in metaMA. The false discovery rate (FDR) was performed for multiple testing corrections of raw *p*-value through the Benjamin and Hochberg method (12, 13). The threshold of DEGs was set at *p*-value < 0.01.

### Functional Enrichment Analysis of DEGs

In order to obtain the biological function and signaling pathways of DEGs, the GeneCoDis3 (http://genecodis.cnb.csic.es/analysis) software was used for Gene Ontology (GO, http://www.geneontology.org/) annotation and Kyoto Encyclopedia of Genes Genomes (KEGG, http://www.genome.jp/kegg/pathway.html) pathway enrichment of DEGs. The threshold of GO function and KEGG pathway of DEGs was all set as FDR < 0.05.

### PPI Network Construction

It is useful to understand the molecular mechanism of bone metastasis from breast cancer by studying the interactions between proteins. In order to gain insights into the interaction between proteins encoded by DEGs and other proteins, the database of BioGRID (http://thebiogrid.org) was used to retrieve the predicted interactions between all proteins encoded by DEGs and other proteins. Then, PPI network was visualized by the Cytoscape Software (http://cytoscape.org/). A node in the PPI network denotes protein, and the edge denotes the interactions.

### Analysis of Potential TFs to DEGs

TFs play crucial roles in regulating gene expression. We downloaded the TFs in the human genome and the motifs of genomic binding sites from the TRANSFAC. Moreover, the 2 KB sequence in the upstream promoter region of DEGs was downloaded from UCSC (http://www.genome.ucsc.edu/cgi-bin/hgTables). Target sites of potential TFs were subsequently distinguished. Finally, the transcriptional regulatory network was visualized by Cytoscape software.

### QRT-PCR *in vitro*

Twenty-five patients with bone metastasis of breast cancer, 25 patients with lung metastasis of breast cancer, and 25 patients with liver metastasis of breast cancer were incorporated in our study. Meanwhile, 25 primary breast cancer patients without metastasis was enrolled, and their adjacent non-cancer breast tissues was considered as normal tissue to be used as control in the present study. Their tumor tissues from metastatic sites were used for RNA isolation. Ethical approval was obtained from the ethics committee of the cancer hospital and informed written consent was obtained from all of subjects.

Total RNA of the tumor tissues from metastatic sites was extracted using the RNA liquid Reagent (TIANGEN) according to the manufacturer's protocols. One microgram RNA was applied to synthesize cDNA by Fast Quant Reverse Transcriptase (TIANGEN). Then real-time PCR was performed in an ABI 7300 Real-time PCR system with SYBR® Green PCR Master Mix (TIANGEN). All reactions were carried out in triplicate. GAPDH was used for the internal reference. Relative gene expression was analyzed by fold change method.

### Receiver Operating Characteristic Analyses

In order to assess the diagnostic value of IBSP, MMP9, TNFAIP6, DHRS3, RIPK4, CSPG4, and CD200 in bone metastasis of breast cancer, receiver operating characteristic (ROC) analyses were performed using pROC package in R language. The area under the curve (AUC) under binomial exact confidence interval was calculated to generate the ROC curve.

### Cell Culture and Transfection With Plasmid and siRNA

Human breast cancer cell line MCF-7 was purchased from ATCC (Manassas, VA, USA) and was cultured in RPMI 1640 medium (Invitrogen, CA, USA) supplemented with 10% fetal bovine serum (Invitrogen, Carlsbad, CA, USA) and incubated at 37°C and 5% CO2. CD200 and TNFAIP6 were selected from up-regulated gene groups and DHRS3, ASS1, and RIPK4 were selected from down-regulated gene groups. Commercial RNA interfering sets targeting CD200 and TNFAIP6 were purchased from Ribobio technology (Shenzhen, Guangdong, China), and negative control of siRNA was also transfected into MCF-7 cells to avoid the influence of transfection itself. Exogenous human full-length DHRS3, ASS1, and RIPK4 plasmid vectors were purchased from Sino biological Inc. (Beijing, China); and empty vector was also transfected into MCF7 cells as negative control. Lipofectamine 2000 (Invitrogen) was used for all transfection studies by following the manufacturer's protocol. Transfection efficiency was examined using qRT-PCR. Briefly, total RNA was extracted from cells using Trizol (Invitrogen, CA, USA) and further purified by RNAeasy mini kit plus DNase I treatment (Qiagen, Germany). The relative mRNA level for each gene was quantified by real-time RT-PCR with SYBR Green (Applied Biosystems, CA, USA), using GAPDH as a control.

### Wound Healing Assay

The migration ability of cells was measured by wound-healing assay. Briefly, 2 × 10^4^ cells were inoculated in 6-cm tissue culture dishes, cultured overnight, scratch was performed when cell growth fusion reached to 90%. Migration images were captured 24 and 48 h after scratching, the migration rate (width) of cells were calculated.

### Transwell Migration

Corning™ BioCoat™ Matrigel™ Invasion Chamber with Corning™ Matrigel Matrix (Thermo Fisher Scientific, Waltham, MA, USA) was used for cell invasion assay. One hundred microliters of cells with concentration of 5 × 10^5^/mL were added in the upper chamber with (8 μm pore), 600 μL medium containing 10% serum was added in the lower chamber. After cultured for 6 h, medium in the chamber were removed and unmigrated cells were swabbed. Cells was fixed by 4% polyformaldehyde for 10 min, stained with crystal violet for another 10 min. The filter membrane was photographed under the inverted microscope (200×) after sealed with the neutral gum. Cells were counted by Image Pro Plus Version 6 software, 3 wells in each group and 5 vision fields of each well were randomly selected to calculate the average number of cells.

## Results

### DEGs Analysis

**Figure 1:**
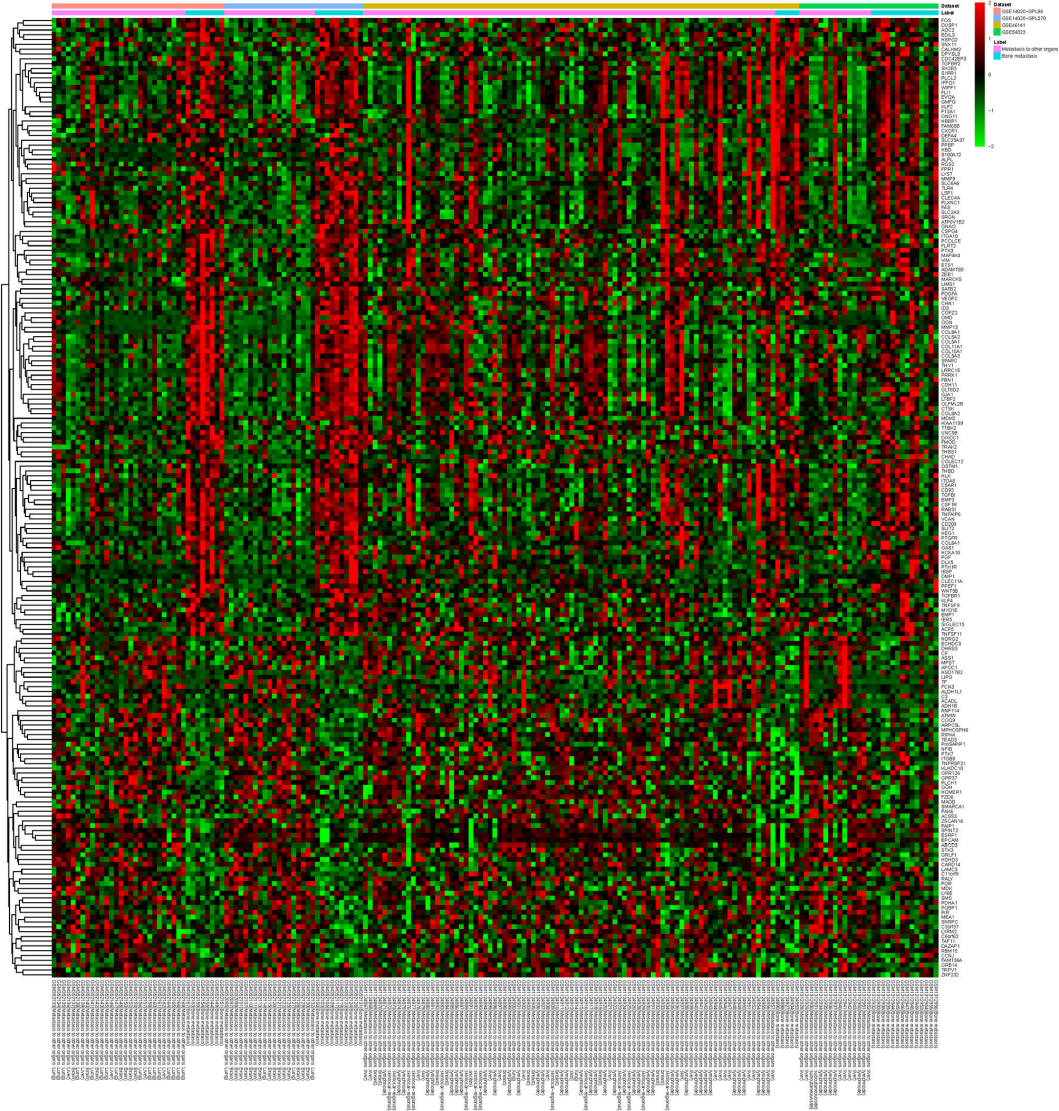
The heat map of top 200 DEGs. Diagram presents the result of a two-way hierarchical clustering of top 200 DEGs and samples. The clustering is constructed using the complete-linkage method together with the Euclidean distance. Each row represents a DEG and each column, a sample. The DEG clustering tree is shown on the right. The color scale illustrates the relative level of DEG expression: red, below the reference channel; green, higher than the reference.

A total of 749 DEGs were identified with the threshold of *p*-value < 0.05, consisting of 469 up-regulated genes and 280 down-regulated genes. Top 10 up-regulated DEGs were integrin binding sialoprotein (IBSP), matrix metallopeptidase 9 (MMP9), matrix metallopeptidase 13 (MMP13), osteomodulin (OMD), cathepsin K (CTSK), TNF alpha induced protein 6 (TNFAIP6), SATB homeobox 2 (SATB2), olfactomedin like 2B (OLFML2B), EGF like repeats and discoidin domains 3 (EDIL3), and procollagen C-endopeptidase enhancer (PCOLCE). The top 10 down-regulated DEGs were dehydrogenase/reductase 3 (DHRS3), argininosuccinate synthase 1 (ASS1), receptor interacting serine/threonine kinase 4 (RIPK4), vimentin (VIM), chondroitin sulfate proteoglycan 4 (CSPG4), ring finger protein 114 (RNF114), NDRG family member 2 (NDRG2), integrin subunit beta 8 (ITGB8), solute carrier family 6 member 6 (SLC6A6), and formyl peptide receptor 1 (FPR1). Detailed information about top 10 up- and down-regulated DEGs was listed in Table 2. The heat map of top 200 DEGs was shown in Figure 1. From the heat map, we can see that these DEGs were distinguished between the bone metastasis and other site metastasis.

**Table 2:** Top 10 up- and down-regulated DEGs.

**Gene ID**	**Gene**	**Bone metastasis**	**Other sites metastasis**	***P*-value**	**FDR**	**Up/down**
3381	IBSP	0.091052	−1.13987	8.66E-15	1.06E-10	Up
4318	MMP9	2.196633	0.711473	4.50E-12	2.74E-08	Up
4322	MMP13	0.044185	−0.65971	3.82E-10	1.55E-06	Up
4958	OMD	0.009911	−0.95916	1.19E-09	3.64E-06	Up
1513	CTSK	1.700623	0.648007	3.23E-09	7.88E-06	Up
7130	TNFAIP6	0.425532	−0.36804	1.81E-08	3.67E-05	Up
23314	SATB2	0.126353	−0.60566	3.32E-08	5.78E-05	Up
25903	OLFML2B	0.748614	0.212792	4.36E-08	6.65E-05	Up
10085	EDIL3	−0.93194	−1.45248	6.10E-08	8.27E-05	Up
5118	PCOLCE	1.030222	0.374014	1.25E-07	0.000151	Up
9249	DHRS3	0.488833	0.944744	5.24E-07	0.000288	Down
445	ASS1	0.439835	1.305644	2.88E-06	0.001086	Down
54101	RIPK4	0.426273	0.68844	3.83E-06	0.001262	Down
7431	VIM	0.928514	1.352016	1.20E-05	0.002822	Down
1464	CSPG4	−0.49526	−0.39281	1.57E-05	0.003477	Down
55905	RNF114	0.739501	1.17908	1.67E-05	0.00364	Down
57447	NDRG2	0.552197	1.037042	2.20E-05	0.004259	Down
3696	ITGB8	−1.35342	−0.49282	4.02E-05	0.006208	Down
6533	SLC6A6	−0.32443	−0.23874	4.86E-05	0.007051	Down
2357	FPR1	−0.56296	−0.17572	8.85E-05	0.01113	Down

*FDR, false discovery rate*.

### Functional and Pathway Enrichment Analyses of DEGs

**Figure 2:**
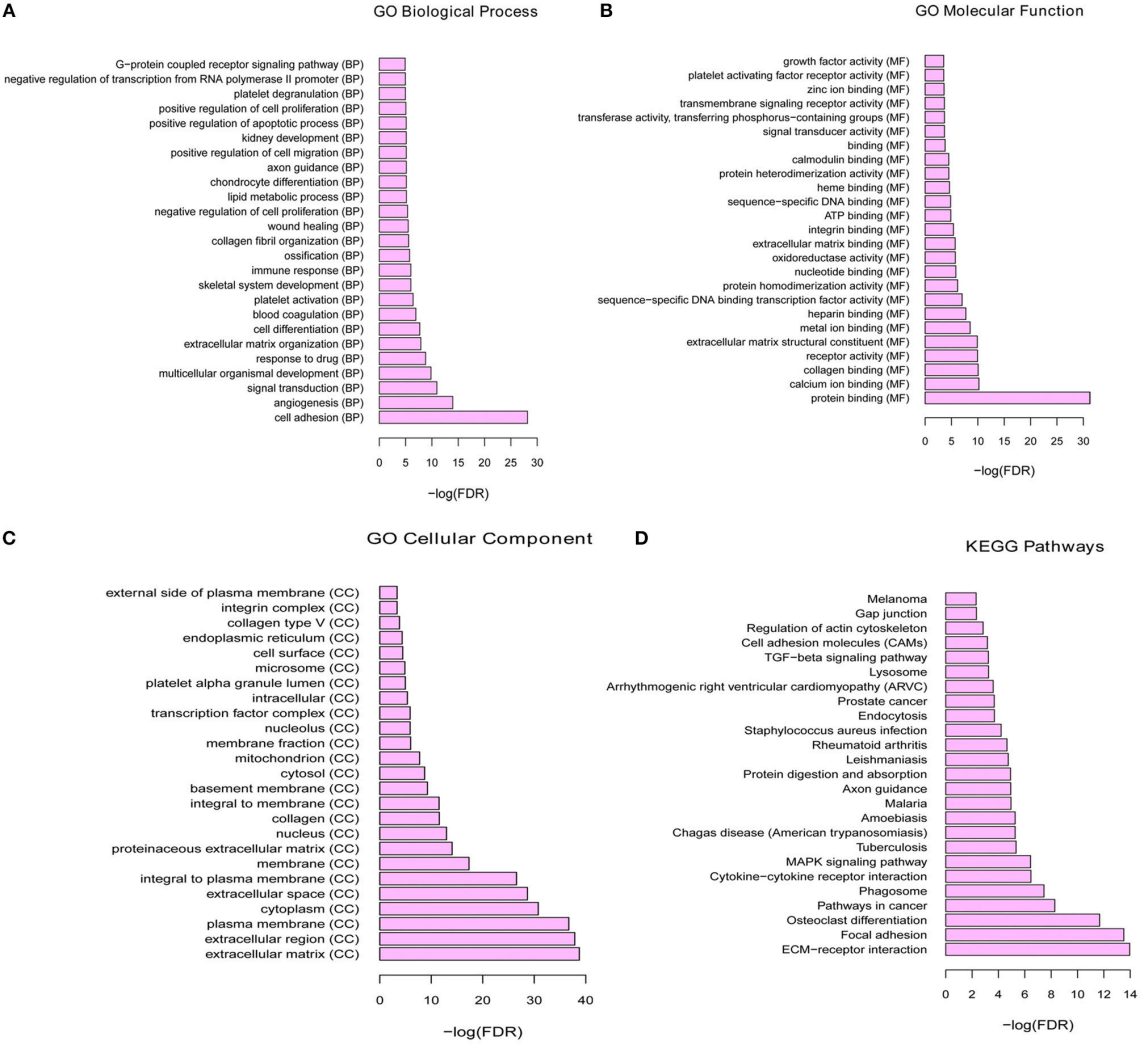
The functional enrichment of GO terms and KEGG pathways for differential mRNA. **(A)** Top 25 significant enrichment biological processes of DEGs. **(B)** Top 25 significant enrichment molecular functions of DEGs. **(C)** Top 25 significant enrichment cellular components of DEGs. **(D)** Top 25 significant enrichment KEGG signal pathways of DEGs.

To investigate the biological function of the identified DEGs in breast cancer bone metastasis, GO term and KEGG pathway enrichment analyses was performed. In GO term and KEGG pathway enrichment analyses, cell adhesion, angiogenesis, and signal transduction were the most significant enrichment in biological process; protein binding, calcium ion binding and collagen binding were the most remark enrichment in molecular function; extracellular matrix, extracellular region, and plasma membrane were the most significant enrichment in cellular component. ECM-receptor interaction, focal adhesion, and osteoclast differentiation were the most significant enrichment in KEGG signaling pathways. Top 25 GO terms of DEGs were presented in Figures 2A–C, respectively. The top 25 KEGG enrichment signal pathways of DEGs were shown in Figure 2D.

### PPI Network

**Figure 3:**
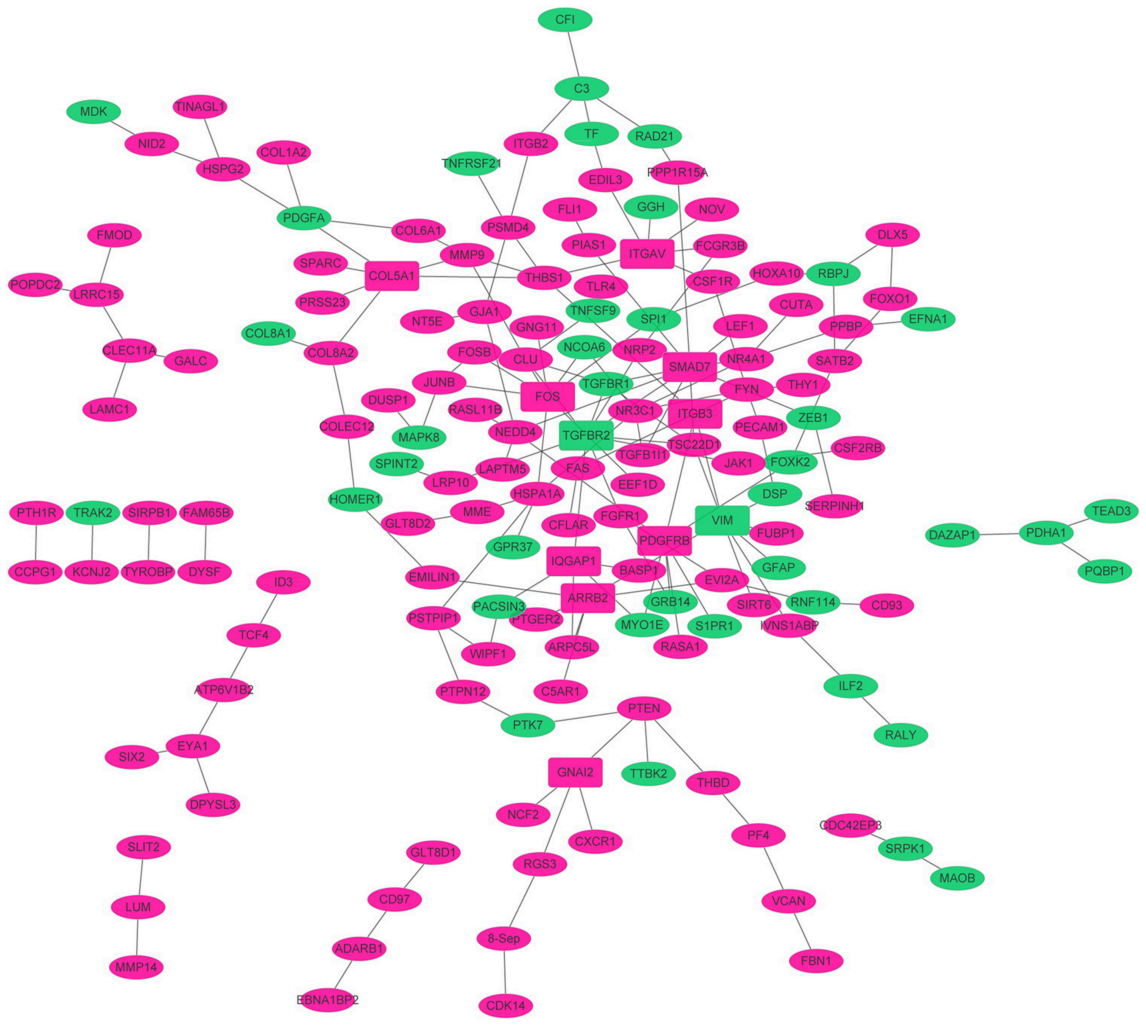
The PPI networks. The node denoted protein, and the edge denoted the interactions. The rose red and green ellipse presented the up- and down-regulated proteins encoded by genes, respectively. The rectangle presented high degree (degree > 5) proteins encoded by DEGs. The solid line and dotted line presented direct interaction and colocalization, respectively.

To obtain the interaction between the proteins encoded by DEGs and other proteins, PPI network was explored and visualized by Cytoscape. PPI networks of proteins encoded by DEGs (FDR < 0.05) and interacting proteins encoded by DEGs (*p* < 0.01) were shown in Figure 3. As shown in Figure 3, the network consisted of 173 nodes and 158 edges. The rose red and green ellipse presented the up- and down-regulated proteins encoded by DEGs, respectively. The rectangle presented DEGs-encoded proteins with high degree (degree > 5). These high degree proteins were SMAD7 (degree = 10), TGFBR2 (degree = 9), VIM (degree = 8), FOS (degree = 8), PDGFRB (degree = 7), COL5A1 (degree = 6), ARRB2 (degree = 6), and ITGAV (degree = 6).

### Establishment of TFs-Target Genes Regulatory Network

**Figure 4:**
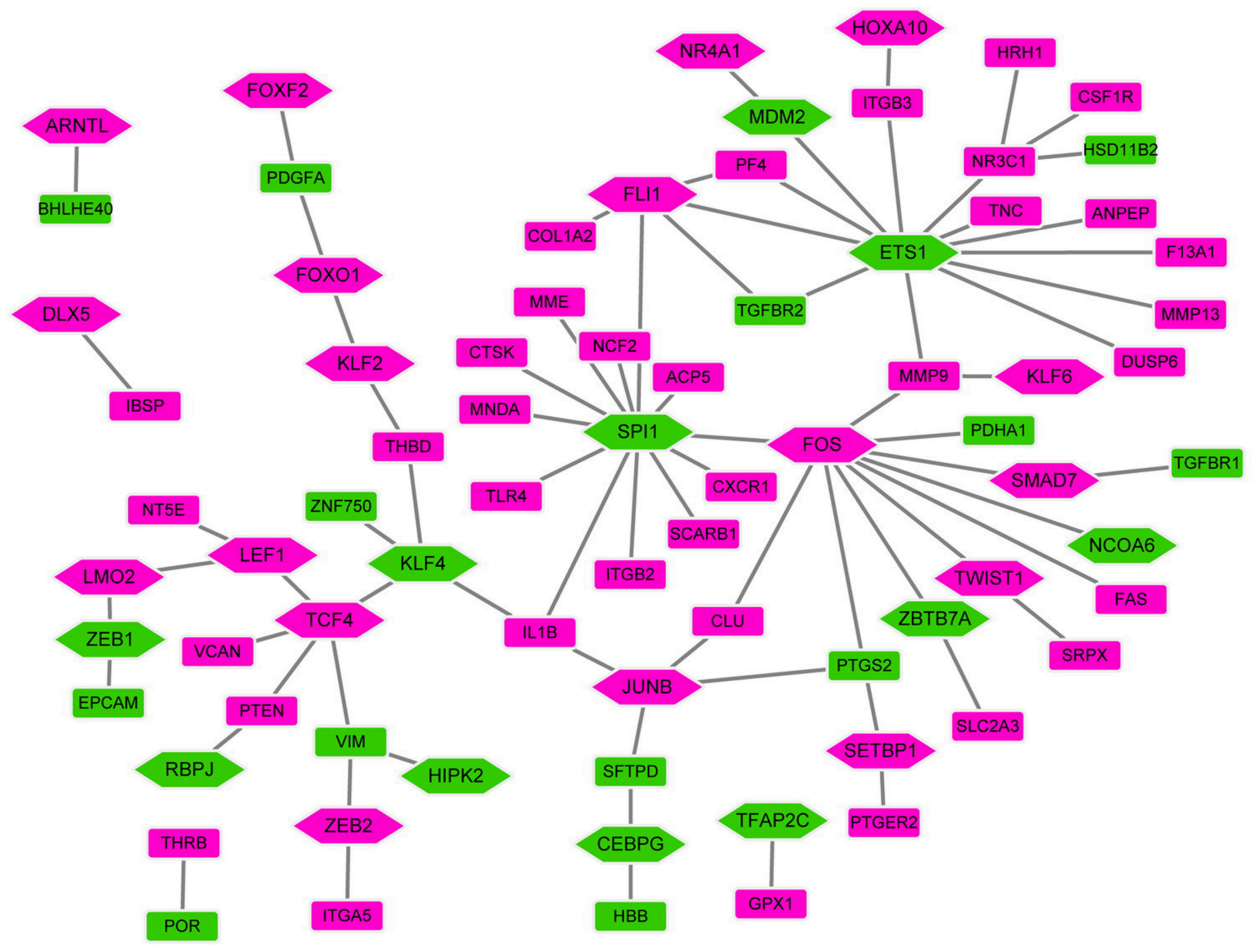
The TFs-target genes networks. In the network, the hexagon and rectangle presented the TFs and DEGs, respectively. The rose red and green ellipse presented the up- and down-regulation, respectively.

In order to investigate the TFs-target genes regulatory network for bone metastasis of breast cancer, we utilized TRANSFAC to obtain 53 TFs (from proteins encoded by all DEGs) regulating other DEGs (Figure 4). In the network, the hexagon and rectangle presented the TFs and DEGs, respectively. The rose red and green ellipse presented the up- and down-regulation, respectively. In the end, we obtained transcriptional regulatory networks consisting of 77 nodes and 77 edges. In this network, the top 7 TFs that covered the most DEGs were ETS1 (degree = 12), SPI1 (degree = 12), FOS (degree = 10), FLI1 (degree = 5), KLF4 (degree = 4), JUNB (degree = 4), NR3C1 (degree = 4).

### QRT-PCR

**Figure 5:**
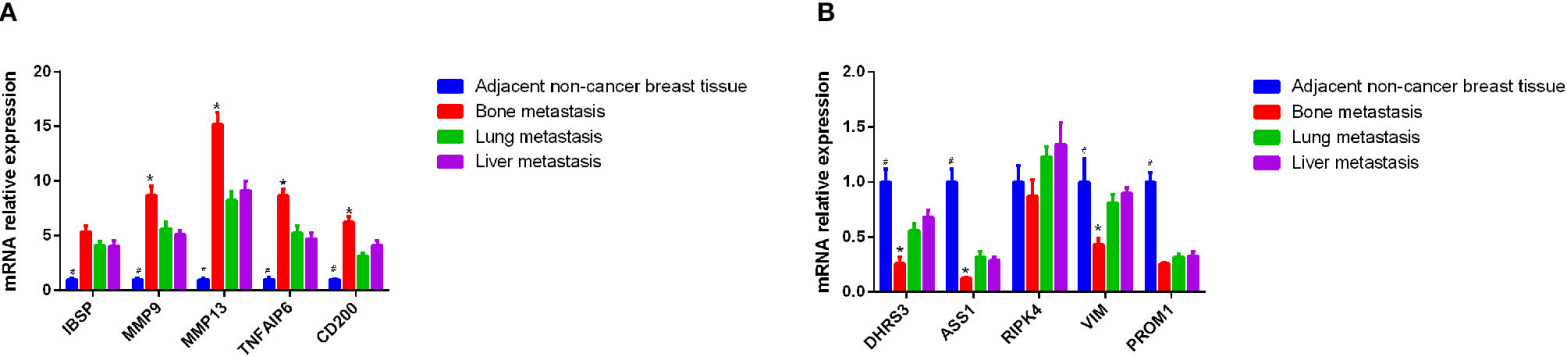
Identification of differential mRNA by QRT-PCR using metastatic bone, liver, and lung tissues. **(A)** Expression of IBSP, MMP9, MMP13, TNFAIP6, and CD200 in the tissue of patients with breast cancer bone metastasis, lung metastasis and liver metastasis by qRT-PCR. The x axis and y axis presented gene name and relative expression, respectively. **(B)** Expression of DHRS3, ASS1, RIPK4, VIM, and PROM1 in the tissue of patients with breast cancer bone metastasis, lung metastasis, and liver metastasis by qRT-PCR. The x axis and y axis presented gene name and relative expression, respectively. ^#^*P* < 0.05, adjacent non-tumor breast tissue compared with three site metastases; *p < 0.05, bone metastasis compare with lung and liver metastasis.

In this study, eight candidate genes were selected from the top 10 up- or down-regulated DEGs, which were IBSP, MMP9, MMP13, TNFAIP6, CD200, DHRS3, ASS1, RIPK4, VIM, and PROM1 for validation of integrated analysis results (Table 2, Figures 5A,B). CD200 and PROM1 was also selected for qRT-PCR validation as cluster of differentiation (CD) markers from DEGs. The relative mRNA expression from adjacent non-cancer breast tissues was normalized as 1. As shown in Figure 5, results showed that the relative expression of MMP9, MMP13, TNFAIP6, and CD200, were significantly up-regulated (*P* < 0.05), while DHRS3, ASS1, and VIM were significantly down-regulated in the bone metastasis compared with lung and liver metastasis (*P* < 0.05). It is noted that, although no statistical significance was found for IBSP, RIPK4, and PROM1, their expression trends were similar with bioinformatics data. All the mRNA level of tested genes from adjacent non-tumor breast cancer was significantly different from metastasis tumor tissues, except RIPK4 (Figure 5).

### ROC Curve Analysis

**Figure 6:**
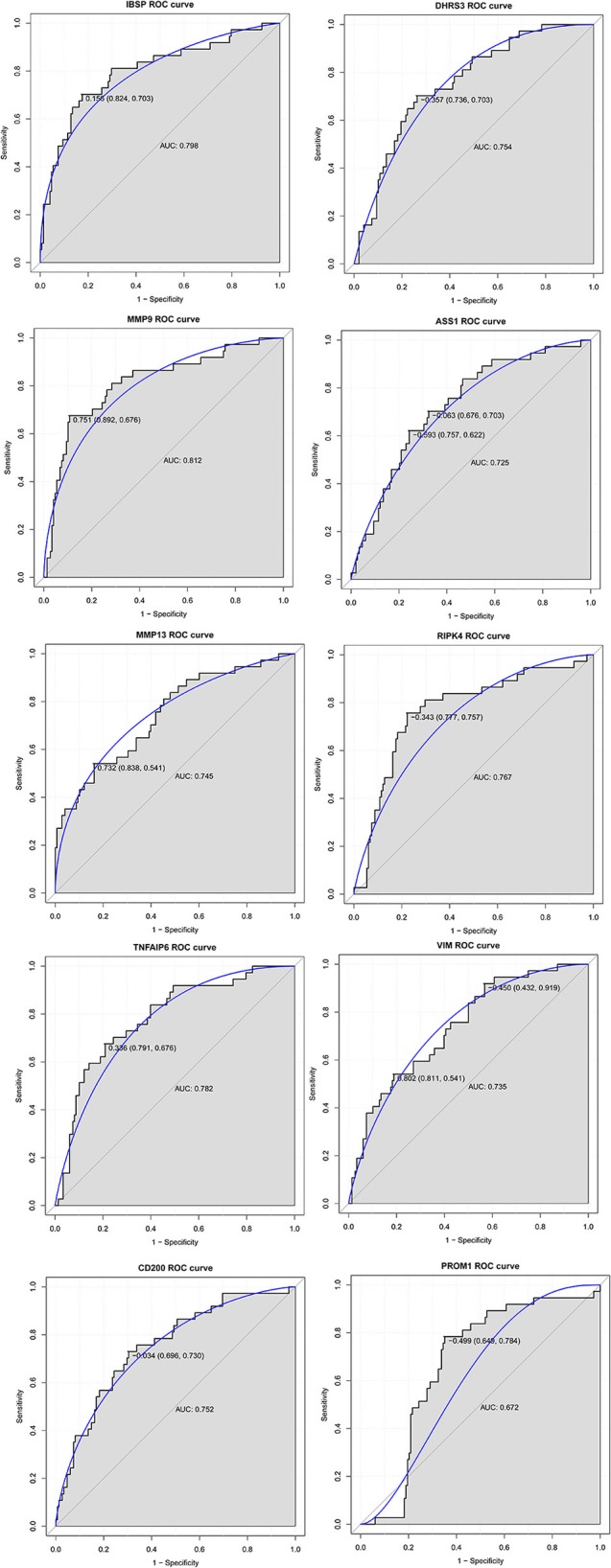
The ROC curve analyses of IBSP, MMP9, MMP13, TNFAIP6, CD200, DHRS3, ASS1, RIPK4, VIM, and PROM1 in breast cancer bone metastasis. The x axis and y axis presented specificity and sensitivity, respectively.

In order to access the discriminatory ability of the above 10 genes used for qRT-PCR validation in breast cancer bone metastasis, ROC curve analyses were conducted and AUC were calculated. As shown in Figure 6, the AUC of these genes was more than 0.7 except PROM1. For breast cancer bone metastasis diagnosis, the sensitivity and specificity of these genes were very applicable.

### Migration and Invasion Ability of Breast Cancer Cell Regulated by Gene Knocking Down or Over-expression

**Figure 7:**
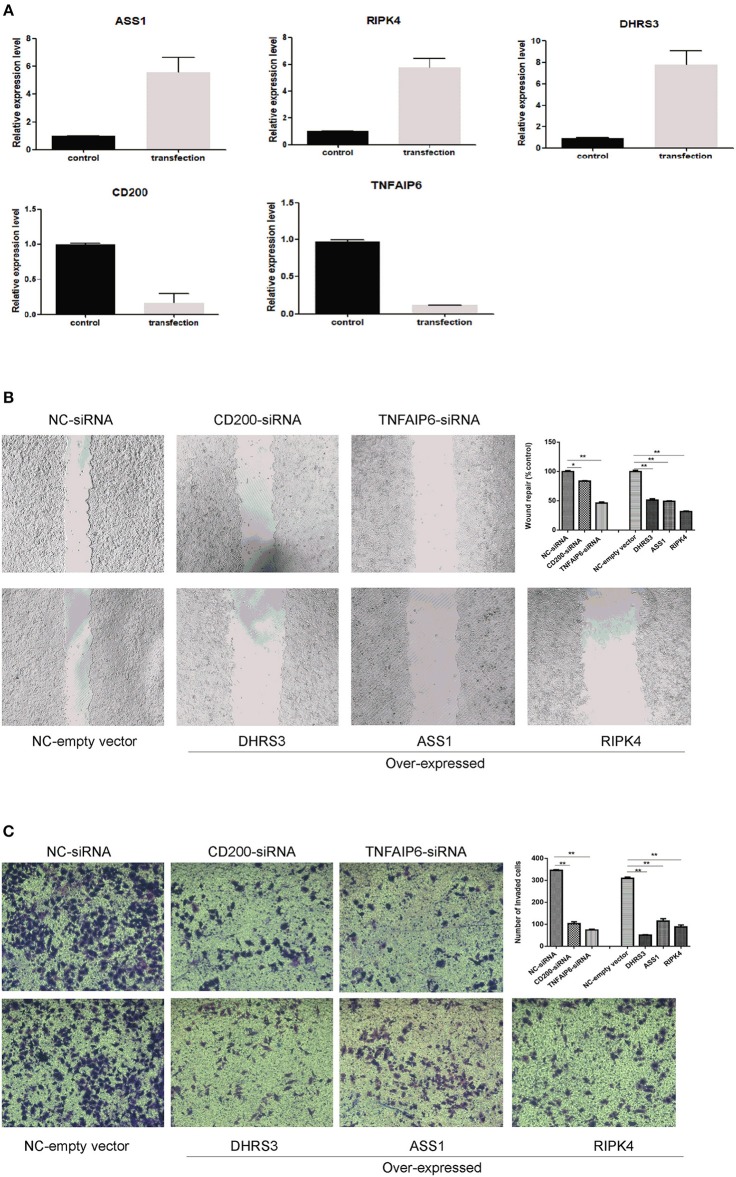
Wound healing assay and transwell invasion assay. **(A)** RT-PCR analysis of selected gene expression in MCF-7 cells after knocking down and knocking-up treatment. **(B)** wound closure was shown at 48 h post scratch (200 × ). Cell migration was assessed by recover of the scratch. The area of the wound was measured at the two time points in every group, and % reduction of initial scratch area was compared with control; and the results were expressed as percentage relative to the corresponding blank control. **(C)** Transwell invasion assay. MCF-7 cells penetrating the membrane were fixed and 0.1% crystal violet stained after 24 h as described in experimental procedures, and cell number penetrating the membrane were counted for each group. *P < 0.05, **P < 0.01. Data were the means of three measurements and the bars represented SD of the mean.

To evaluate the impact of these predicted genes on cell migration and invasion, the wound healing assay and matrigel invasion assay were employed. As shown in Figure 7A, by RT-PCR, we confirmed that RNA interference significantly reduced the mRNA level of CD200 and TNFAIP6, and meanwhile exogenous expressions of DHRS3, ASS1, and RIPK4 were remarkably higher than empty-vector control cells. We found that overexpression of DHRS3, ASS1, and RIPK4 inhibited MCF-7 cell migration, as well as knock-down CD200 and TNFAIP6 (Figure 7B). Consistent with this finding, matrigel invasion assay showed that DHRS3, ASS1, and RIPK4 overexpression significantly reduced invasion capacity of MCF7, which is similar as knocking down CD200 and TNFAIP6 (Figure 7C). These observations suggested that these predicted gene played important roles in regulating migration and invasive potential of breast cancer cells.

## Discussions

Among numerous malignancies, metastatic breast cancer is the second leading cause of woman death (14). Clinically, breast cancer can metastasize to various sites such as brain, liver, lung, and bone, and bone is the most common metastasis site (15). Therefore, maintaining bone health is an important clinical challenge in breast cancer patients. In this study, we found several DEGs and signaling pathways related to breast cancers bone metastasis, which may be helpful in understanding the bone metastasis mechanism of breast cancer.

In this study, a total of 749 DEGs were identified, consisting of 469 up-regulated genes and 280 down-regulated genes and eight DEGs were selected from the top 10 up- or down-regulated DEGs, such as IBSP, MMP9, MMP13, TNFAIP6, DHRS3, ASS1, RIPK4, and VIM for the following qRT-PCR validation. CD200 and PROM1 was also selected for qRT-PCR validation as cluster of differentiation markers. Although there is no statistical difference except CD200 and DHRS3, the tendency of expression was generally consist with the bioinformatics analysis results. In addition, their diagnostic ability was also evaluated, and it appeared that IBSP, MMP9, MMP13, TNFAIP6, CD200, DHRS3, ASS1, RIPK4, and VIM may be considered as specific prognostic markers for bone metastasis to identify primary breast cancers that have potential to metastasize to bone tissue.

There are some preclinical evidences of factors associated with breast cancer cells homing to bone tissue. Awolaran et al. pointed out that 15 proteins expressed by breast cancer cells mediated breast cancer bone metastasis: ICAM-1, CDH11, osteoactivin, bone sialoprotein, CCN3, IL-11, etc. (16). In a study by Mercatali et al., SPARC, IBSP, MMP9 was found to be over expressed in patient-derived xenograft of breast cancer bone metastasis compared to cell lines of MCF7 and MDA-MB-231 (17).

In our study, we detected some published potential biomarker of breast cancer bone metastasis, or their function has been reported in breast cancer bone metastasis such as SPARC, IBSP, MMP9, MMP13. SPARC was found as a DEG in breast cancer bone metastasis, while wasn't in the list of the up- or down-regulated DEGs. SPARC, also known as osteonectin, is involved in the chemoattraction of both breast and prostate cancer, and has been detected as a biomarker for bone metastasis (18).

IBSP, an osteoblast differentiation marker, is associated with osteoblastogenesis, bone formation and tumor-induced osteolysis (19, 20). It is reported that IBSP is up-regulated in luminal B2 breast cancer and breast cancer with bone metastasis (17), while the expression level of IBSP was relatively low in older patients with breast cancer compared with those young patients (21, 22). Furthermore, the expression of IBSP is associated with development of metastasis and poor prognosis in breast cancer (23, 24).

Matrix metallopeptidase 9 (MMP9) and matrix metallopeptidase 13 (MMP13), members of the peptidase M10 family of matrix metalloproteinases (MMPs), is normally expressed by resident bone cells primarily by osteoclasts and osteoblasts (25). It was no surprise to find high levels of it in the bone metastasis. MMP9 and MMP13 is up-regulated in breast cancer tissue, and were involved in bone metastasis of tumor progression (26–28). It is reported that up-regulated MMP9 (29) and MMP13 is both associated with poorer overall survival and can serve as a prognosis indicator of breast cancer (30, 31).

Besides some published potential biomarkers of breast cancer bone metastasis were identified in our study, we also detected some novel biomarkers or their relationship with breast cancer bone metastasis has never been reported, such as TNFAIP6, DHRS3, ASS1, RIPK4, VIM, CD200. However, their function in breast cancer has been investigated except RIPK4.

Cancer cells can create an inflammatory microenvironment to enhance the tumor metastatic process (32). It is reported that excessive and chronic activation of the immune system is involved in breast cancer metastasis (33). TNF alpha induced protein 6 (TNFAIP6) encodes inflammatory response factors and regulates anti-inflammation response and immunosuppression (34, 35). It is indicated that TNFAIP6 is a prognostic-related cytokine in breast cancers (36).

Dehydrogenase/reductase 3 (DHRS3) has been found to be involved in the tumor suppressive pathway and constitutively expressed in breast cancer cell lines (37, 38). It is found that DHRS3 is down-regulated in breast cancer and breast cancer brain metastases (39, 40). CD200 molecule (CD200) serum levels are significantly higher in the early and the advanced stage breast cancer patients (41). Gorczynski et al. found that over expression of CD200 increased breast cancer lymph node metastasis (42). In addition, it has been demonstrated that the enhancement of the CD200-CD200 receptor interaction inhibits the metastasis of breast cancer (43). It is reported that CD200 analogs may have therapeutic potential in treating aggressive breast carcinoma (33). ASS1 (44) and VIM (45) are both prognostic indicators in breast cancer patients. The research of TNFAIP6, DHRS3, ASS1, RIPK4, VIM, and CD200 in breast cancer may indicate that those genes may play a crucial role in the development of breast cancer bone metastasis.

For RIPK4, their relationship to breast cancer still remains unreported. Receptor interacting serine/threonine kinase 4 (RIPK4) is involved in a number of signaling pathways and plays an important role in regulating cells proliferation, cell differentiation and cell apoptosis (46). More and more evidences have shown that RIPK4 is up-regulated in tumor tissues and promotes the occurrence and progression of cervical cancer and ovarian cancer (47, 48).

According to the KEGG pathway enrichment analysis, we found two important signaling pathways including osteoclast differentiation and rheumatoid arthritis in the bone metastasis of breast cancer. Bone metastasis of breast cancer induces severe osteolysis with increased bone resorption (25). Interestingly, osteoclast differentiation is involved in the process of bone resorption. It is reported that bisphosphonate, an inhibitor of osteoclastic bone resorption, is a valuable agent in treating bone metastasis of breast cancer patients (49). Previous epidemiologic study indicated that breast cancer patients with rheumatoid arthritis had poor prognoses and higher mortality compared with breast cancer patients without rheumatoid arthritis (50). In identified DEGs, we found two high degree gene in both PPI and TF-target gene networks, which were fos proto-oncogene, AP-1 transcription factor subunit (FOS) and one of top 10 up-regulated gene cathepsin K (CTSK) was involved in osteoclast differentiation and rheumatoid arthritis. FOS is an important osteoclasts' differentiation maker involved in osteoclastogenesis (51). Sotiriou et al. found that FOS was a confident driver gene for breast cancer metastasis (52). CTSK is an osteoclast phenotypic marker. It is found that CTSK is secreted into the isolated environment, which leads to the bone resorption (53). Le Gall et al. found that CTSK inhibitors have been used in the preclinical studies, which have shown the effectiveness of CTSK in the reduction of breast carcinomas bone metastases in experimental animal model (54).

To further confirm the results of bioinformatics prediction, the wound healing assay and matrigel invasion assay were performed. Total five genes were selected, two are up-regulated in breast cancer and three are down-regulated in breast cancer. As shown in Figure 7, altering expression of these genes in breast cancer cells significantly influencing the migration and invasion ability.

In summary, we found several DEGs including IBSP, MMP9, MMP13, TNFAIP6, DHRS3, ASS1, RIPK4, VIM, CD200, and PROM1 may play important roles in the process of bone metastasis from breast cancer. Among which, IBSP, MMP9, TNFAIP6, DHRS3, RIPK4, and CD200 had a diagnose value for patients with breast cancer bone metastasis. Additionally, osteoclast differentiation and rheumatoid arthritis signaling pathways and related DEGs including FOS and CTSK may be involved in the development of bone metastasis from breast cancer. However, there are limitations to our study. Firstly, sample size in the QRT-PCR was small and a larger-scale study is further needed. Secondly, the biological function of identified DEGs wasn't investigated. Experiments in animal model and cell culture are further needed to explore underlying functional mechanism of breast cancer bone metastasis.

## Author Contributions

YZ is in charge of data collection and data mining. WH is in charge of molecular biological experiments and revising manuscript. SZ is in charge of data analysis, molecular biological experiments, and manuscript draft.

### Conflict of Interest Statement

The authors declare that the research was conducted in the absence of any commercial or financial relationships that could be construed as a potential conflict of interest.
